# Vasculogenic Cytokines in Wound Healing

**DOI:** 10.1155/2013/190486

**Published:** 2013-02-28

**Authors:** Victor W. Wong, Jeffrey D. Crawford

**Affiliations:** Department of Surgery, Oregon Health and Science University, 3181 SW Sam Jackson Park Road, Portland, OR 97239, USA

## Abstract

Chronic wounds represent a growing healthcare burden that particularly afflicts aged, diabetic, vasculopathic, and obese patients. Studies have shown that nonhealing wounds are characterized by dysregulated cytokine networks that impair blood vessel formation. Two distinct forms of neovascularization have been described: vasculogenesis (driven by bone-marrow-derived circulating endothelial progenitor cells) and angiogenesis (local endothelial cell sprouting from existing vasculature). Researchers have traditionally focused on angiogenesis but defects in vasculogenesis are increasingly recognized to impact diseases including wound healing. A more comprehensive understanding of vasculogenic cytokine networks may facilitate the development of novel strategies to treat recalcitrant wounds. Further, the clinical success of endothelial progenitor cell-based therapies will depend not only on the delivery of the cells themselves but also on the appropriate cytokine milieu to promote tissue regeneration. This paper will highlight major cytokines involved in vasculogenesis within the context of cutaneous wound healing.

## 1. Introduction

It is estimated that diabetic and cardiovascular complications will account for $9 trillion in US healthcare costs over the next thirty years [1]. These complications are often associated with impaired blood vessel growth in response to tissue hypoxia and ischemia. Chronic nonhealing wounds represent an important public health problem as populations prone to impaired wound healing continue to grow (e.g., diabetics, elderly, and obese) [2]. The estimated healthcare cost of diabetic foot ulcers alone has been estimated at $45,000 per patient [3, 4]. Thus, strategies to augment the neovascularization response to injury may dramatically improve the quality of life for these patients and significantly reduce the global biomedical burden [1, 5, 6]. 

Regulation of blood vessel development in response to tissue injury or ischemia is critical for maintenance of healthy tissues [7]. A robust vascular response to deliver immune cells and metabolic substrates is important for cutaneous wound healing [8]. In addition, coordinated neovascularization programs are essential for normal organ development during embryogenesis [9]. Conversely, dysregulated signaling can promote tumor growth and metastasis [10, 11]. A better understanding of blood vessel formation in both health and disease states may result in more effective therapies for a wide range of diseases.

**Figure 1:**
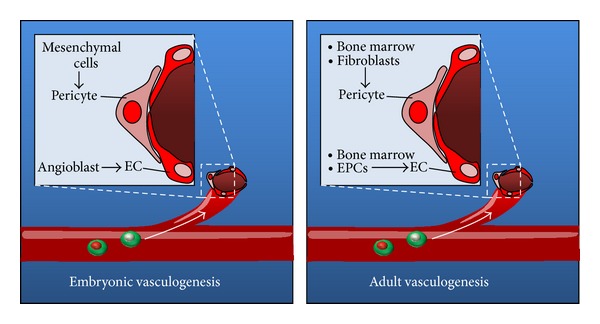
Embryonic versus adult vasculogenesis. During embryonic vascular development, endothelial cells (EC) derived from angioblast precursors migrate to regions of neovessel formation. Additionally, mesenchymal stem cells (MSCs) differentiate into pericytes which support and guide the development of endothelial cells. In adult tissues, vasculogenesis proceeds via recruitment of endothelial progenitor cells (EPCs) to neovessels. Supporting pericytes are thought to be derived from local fibroblasts or bone-marrow-derived mesenchymal cells. These complex interactions are mediated by cytokine networks responsible for creating functional three-dimensional vascular systems during development and throughout life.

During embryogenesis, mesoderm-derived angioblasts organize to form blood vessels via *vasculogenesis* [12]. It was initially believed that all subsequent blood vessel growth occurred through sprouting of preexisting endothelial cells via *angiogenesis* [13]. However, it is now known that the vascular programming present during embryonic development is recapitulated in various postnatal states during a process known as adult* vasculogenesis* [14] (Figure 1). Vasculogenesis plays a critical role in maintaining tissue homeostasis throughout the body [15]. Disruption of these pathways can sustain pathogenic processes (e.g., in skin, heart, kidney, and brain) that are only starting to be appreciated on a molecular level. The remainder of this paper refers to postnatal vasculogenesis and focuses on major vasculogenic cytokines in the clinical context of wound healing. 

## 2. Endothelial Precursor and Other Provasculogenic Cells

Endothelial precursor cells (EPCs) are bone-marrow-derived progenitor cells that participate in vasculogenesis and were first identified by Asahara et al. [16]. These cells are recruited to sites of ischemia and divide to form syncytial masses which tubularize and canalize to form a patent vascular network [17]. Although the molecular identification of EPCs remains a topic of debate, studies suggest that two functionally distinct subpopulations exist based on *in vitro* isolation techniques: early outgrowth EPCs and late outgrowth EPCs [18, 19]. Specifically, early outgrowth EPCs appear to function in a paracrine role in promoting neovascularization whereas late outgrowth EPCs directly differentiate into endothelial tubules [19]. Transcriptional and proteomic profiling of these populations suggests that early outgrowth EPCs may be of monocytic origin and restricted in their ability to promote neovascularization clinically [20].

EPCs have also been characterized based on their surface expression profiles [21]. In human studies, combinations of surface markers used to identify EPCs often include CD34+, CD133+, and VEGFR-2+. In mice, common EPC surface markers include Sca-1+, Lin-, Flk-1+, and cKit+. It is important to note that none of the markers used are specific for EPCs. Regardless of how they are classified, a common feature of EPCs is their ability to mobilize and home to injured areas and promote vessel formation [22]. Various signaling molecules are highly implicated in this process and include transforming growth factor beta (TGF*β*) and matrix metalloproteinase-9 (MMP-9) [23].

EPCs are thought to mobilize from the bone-marrow or other tissues and home to areas of endothelial damage via adhesion molecules. The secreted proteases cathepsin L and MMP2 regulate the transmigration of EPCs, which subsequently mature and differentiate towards the endothelial lineage [24, 25]. Nitric oxide signaling and reactive oxygen species have also been implicated in EPC activity, potentially affecting their colony-forming potential and ability to counteract ischemic stress [26]. Nitric oxide pathways have even been linked to the ability of hormonal estrogens to promote EPC proliferation and mobilization [27].

**Figure 2:**
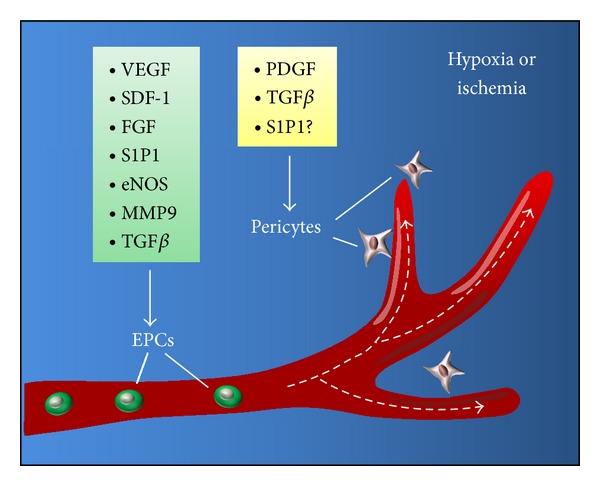
Critical cytokines implicated in vasculogenesis. Signaling molecules such as vascular endothelial growth factor (VEGF), stromal derived factor-1 (SDF-1), fibroblast growth factor (FGF), sphingosine-1-phosphate (S1P), endothelial nitric oxide synthase (eNOS), matrix metalloproteinase-9 (MMP9), and transforming growth factor *β* (TGF*β*) regulate the function of EPCs during vasculogenesis. Pericyte activity during vasculogenesis appears to be modulated by platelet-derived growth factor (PDGF), TGF*β*, and possibly S1P.

Another cell population intimately involved in vascular morphogenesis is the pericyte, a supportive stromal-like cell that retains the pluripotency of mesenchymal stem cells (MSCs) [28]. They reside at the interface between endothelial cells and the surrounding tissue, producing proangiogenic signals that regulate endothelial cell differentiation and growth [29]. Through both direct physical interaction and paracrine signaling, endothelial cells and pericytes engage in complex crosstalk that is essential for normal adult vasculogenesis [30] (Figure 2). Fibroblasts have also been shown to facilitate EPC migration, branching, and sprouting in collagen matrices *in vitro*, potentially via cytokine signaling [31]. Finally, platelets are synergistically involved in vasculogenesis, elaborating potent cytokines that regulate the recruitment and differentiation of EPCs [32]. Diverse cell types are clearly involved in the formation of new blood vessels and the cytokine networks through which they communicate play a critical role in the tissue response to injury. 

## 3. Major Vasculogenic Cytokines (Table 1) 

### 3.1. VEGF

Vascular endothelial growth factors (VEGF) are a family of cytokines important in both embryonic and post-natal vascular development [33]. They play a crucial role in endothelial cell motility, proliferation, and survival [34]. This wide range of effects is mediated in part by the multiple VEGF subtypes and the associated family of VEGF receptor (VEGFR) protein tyrosine kinases. Five human VEGF isoforms (A, B, C, D, and placental growth factor-PlGF) are produced by differential splicing of VEGF mRNA. VEGF-A is involved in vascular growth, lymphatic development, and vascular malformations [35]. The role of VEGF-B in vascular development is poorly understood but may be associated with blocking apoptosis [36]. VEGF-C and VEGF-D are involved in lymphangiogenesis, and PIGF appears to regulate angiogenesis, wound repair, and inflammation [35].

 VEGF-A has been shown to promote adult vasculogenesis via bone-marrow-derived EPC mobilization [37], a process that acts via VEGFR1 and VEGFR2 in a tumor model [38]. In a small animal model of soft tissue ischemia, VEGF levels and circulating VEGFR2+ cells were increased following injury, findings that correlated with migration of EPC populations to ischemic tissue [14]. Furthermore, it has been shown that topical VEGF delivery can improve diabetic wound healing in a murine model through local upregulation of angiogenic cytokines and recruitment of bone-marrow-derived vasculogenic cells [39].

VEGF has also been shown to regulate the expression of endothelial cell surface proteins known as integrins that link cells with the extracellular matrix. Integrins, which comprise a family of transmembrane heterodimeric proteins, play a major role in controlling EPC mobilization and homing to areas of tissue injury and ischemia [40]. Specifically, integrin *α*5*β*1 has been shown to promote VEGF-induced differentiation of EPCs *in vitro*, highlighting the importance of both structural and cytokine signals in regulating EPC activity following injury. 

A key mechanism that regulates VEGF expression is the hypoxia-inducible factor-1 (HIF-1) pathway. HIF-1 is a transcription factor that exists as a dimeric complex consisting of a cytoplasmic *α* subunit and a nuclear *β* subunit [41]. In the setting of hypoxia, HIF-1*α* (which is degraded under normoxic conditions) translocates into the nucleus to complex with HIF-1*β*, initiating the transcription of neovascularization genes including VEGF. Studies have demonstrated that impaired HIF-1*α* binding to its coactivator p300 may underlie diabetic impairments in wound healing [42]. Thus, strategies to stabilize HIF-1*α* may enhance EPC mobilization and function [43] and have been shown to improve cutaneous wound healing in diabetic mice [42, 44]. 

### 3.2. SDF-1

Stromal cell-derived factor-1 (SDF-1) is a chemokine which plays a crucial role in EPC and hematopoietic stem cell (HSC) trafficking through the circulation [41]. SDF-1 binds exclusively to the chemokine receptor CXCR4, which is expressed by circulating cells and regulates their recruitment from bone-marrow [45]. In addition, SDF-1 mediates the activation of circulating stem cells during embryonic organogenesis and vascular development [46], suggesting that it may serve similar functions in post-natal neovascularization. Dysfunctional SDF-1 pathways have been highly implicated in aged and diabetic wound healing in preclinical models [47], underscoring the importance of chemokine-mediated signaling networks in normal wound healing.

Researchers have examined the role of SDF-1 in peripheral vasculogenesis and tissue repair. It has been demonstrated that SDF-1 gene expression in EPCs is regulated by the transcription factor HIF-1*α* and that cutaneous tissues express SDF-1 in response to hypoxia [48]. In addition, blockage of either SDF-1 or its receptor CXCR4 can prevent stem cell recruitment to ischemic tissues. Local delivery of SDF-1 into ischemic muscle has been shown to enhance vasculogenesis via EPC recruitment [49], highlighting its potential as a therapeutic chemokine. Additionally, plasmid gene transfer of SDF-1 has been demonstrated to augment neovascularization through VEGF [50]. In the setting of diabetic wound healing, administration of SDF-1 is capable of reversing the impairment in EPC homing to injured tissue [51]. Other forms of tissue injury have also been shown to activate SDF-1. In a mouse burn wound model, researchers have characterized SDF-1 expression in the healing margin of burn wounds [52]. Ionizing radiation injury also appears to stimulate vessel formation via SDF-1, however in a HIF-independent manner [53]. Together, these studies collectively highlight the importance of SDF-1 in regulating wound vasculogenesis and suggest a role for chemokines in the treatment of chronic wounds.

### 3.3. PDGF

The platelet-derived growth factor (PDGF) family of ligands and receptors is closely related to VEGF and may have evolved from a common gene [54]. The family of four ligands (PDGF-A, PDGF-B, PDGF-C, and PDGF-D) assemble intracellularly and undergo transcriptional and posttranslational modifications. Specifically, the homodimer PDGF-BB recruits perivascular cells during vasculogenesis, possibly through the generation of reactive oxygen species and subsequent activation of extracellular-regulated kinase 1, 2 (ERK 1, 2) [55]. Endothelial-derived PDGF-BB also induces progenitor cell migration and expansion during vascular development [56] and is critical during vascular bed formation by mesangial progenitor cells [57]. VEGF ligands can also bind and activate PDGF pathways, a process important during MSC-associated vasculogenesis [58].

 Researchers have exploited PDGF pathways to control neovascularization in various animal models. For example, nanofibrous scaffolds incorporated with PDGF have been shown to activate cytokine signaling and improve angiogenesis during wound repair in rats [59]. Additionally, a constitutively activating mutation of the PDGF receptor was introduced into embryonic stem cells and shown to enhance vascular development both *in vivo* and *in vitro*, potentially through VEGF pathways [60]. Furthermore, a PDGF-receptor antagonist has been shown to inhibit human tumor growth in a rat model, an effect that was augmented using anti-VEGF antibody [61]. 

In human studies, neovessels in revascularized wounds exhibit strong PDGF receptor staining [62]. These findings are consistent with data demonstrating that PDGF is a primary mediator of vessel maturation [63]. PDGF-dependent pathways are thought to drive angiogenic sprouting and vessel enlargement via vascular cell migration and proliferation [56]. In fact, PDGF was the first growth factor to be approved by the United States Food and Drug Administration for the clinical treatment of ulcers [64]. Taken together, these studies indicate that PDGF signaling is closely associated with VEGF pathways and is important during both developmental and adult vasculogenesis.

### 3.4. FGF

The fibroblast growth factor (FGF) family of cytokines displays diverse functional properties that are important in multiple aspects of wound repair including vasculogenesis [65]. Although FGF and VEGF differentially activate genes and stimulate the development of different vessel types, FGF appears to induce a vasculogenic response that is highly dependent on VEGF [66]. In myocardial tissues, FGF-2 has been shown to augment angiogenesis and vascular remodeling in response to ischemic injury [67]. FGF-1 has been used to induce neovascularization in both an omentum model and a vascular pedicle model in rats [68, 69], suggesting that FGF-based strategies may be effective in promoting blood vessel formation in complex tissue constructs. Recently, researchers demonstrated improved neovascularization in a murine hindlimb ischemia model using an FGF-based hydrogel delivery system [70].

 EPCs express receptors for FGF and a subpopulation of CD34-expressing HSCs that specifically expresses FGFR-1 has been shown to differentiate into endothelial cells *in vitro* [71]. FGF-1 has been shown to regulate the proliferation and differentiation of EPC-like mesenchymal cells [72], suggesting it may control neovascularization mediated by endothelial-stromal cell interactions. FGF also appears to function via autocrine and paracrine mechanisms in endothelial cells [73] and may play a role in tumor angiogenesis and invasiveness [74]. Recently, researchers topically applied EPCs to diabetic wounds in mice and detected increased local expression of FGF and VEGF which corresponded with improved wound healing and vascularization [75], supporting a key role for these cytokines in vasculogenesis during soft tissue repair.

### 3.5. GM-CSF

Granulocyte macrophage colony stimulating factor (GM-CSF) is a potent cytokine that stimulates the mobilization of hematopoietic progenitor/myeloid cells and nonhematopoietic cells (e.g., bone-marrow MSCs) [76]. During wound healing, multiple cell types including keratinocytes, fibroblasts, macrophages, endothelial cells, dendritic cells, and lymphocytes secrete GM-CSF. It has been shown to directly promote reepithelialization and induce secondary cytokine secretion from various wound healing cells [77]. Clinical trials have demonstrated the efficacy of topically applied recombinant human GM-CSF for deep partial thickness burn wounds, highlighting the importance of this cytokine in human wound repair [78].

GM-CSF is related to interleukins (IL)-3 and IL-5 and plays diverse roles in homeostasis and disease [79]. Its role in angiogenesis is partly mediated by monocytes and VEGF-associated pathways [80]. In human endothelial cells, GM-CSF activates intracellular phosphatidyl-inositol-3-kinase and Jak/Stat signaling during vascular tubule formation *in vitro*. Interestingly, immune defense pathways have been associated with GM-CSF-stimulated angiogenesis and may represent an integrated mechanism for tissue defense and regeneration following injury [81].

GM-CSF has also been closely linked to vasculogenic processes. It has been shown to stimulate EPC tubule formation, proliferation, migration, and viability in a dose- and time-dependent manner, effects which were mediated in part by ERK signaling and upregulation of VEGF and integrin *β*2 [82]. GM-CSF has also been shown to enhance EPC recruitment and vasculogenesis in murine and rabbit hindlimb ischemia models [76], potentially via direct activation of endothelial cells during neovascularization [83]. GM-CSF pathways have also been implicated in tumor vasculogenesis [84], indicating that it regulates blood vessel formation in both health and disease states. 

### 3.6. S1P

Sphingosine-1-phosphate (S1P) is a sphingolipid metabolite found in high concentrations in blood and implicated in vascular development. It is secreted most prominently by platelets, suggesting that it may have an important role in tissue repair. Further, it has been shown to act via distinct receptor pathways to regulate keratinocyte and fibroblast chemotaxis, processes that are critical for normal wound healing [85, 86]. As proof of concept, subcutaneous injections of S1P were able to significantly improve diabetic wound healing and neovascularization in rodent models [87]. 

Gradients in S1P levels are known to mediate the migration of endothelial cells, potentially through a recently identified S1P transporter (SPNS2) [88]. S1P is thought to stabilize vasculature in part through regulation of VEGF pathways and cadherins junctions, processes potentially altered by blood flow mechanotransduction signaling [89]. Cadherin and S1P pathways have also been linked to vascular development in a zebrafish model [90], suggesting a key role for S1P in maintaining vascular integrity.

During embryonic vasculogenesis in mice, S1P has been demonstrated to promote migration of angioblasts and endothelial cells [91]. S1P pathways have also been implicated in blood vessel development. For example, mice lacking the receptor for S1P displayed immature vessels that lacked pericytes and smooth muscle elements [92]. Furthermore, other growth factors such as PDGF may act through sphingolipid signaling to promote cellular motility during blood vessel development [93], highlighting the functional diversity of this signaling pathway in vascular biology.

### 3.7. MMP-9 and Other Proteases

MMP-9 is a soluble extracellular protease that plays diverse roles in wound repair. Paradoxically, high levels of MMP-9 have been implicated in chronic nonhealing wounds as well as scarless wound repair in athymic mice [94, 95]. However, mice that lack MMP-9 also exhibit delayed wound healing with disordered collagen remodeling, suggesting that tight regulation of this protease is critical for normal cutaneous repair and remodeling [96]. Recent studies suggest that keratinocyte secretion of MMP-9 may be crucial to maintain normal basement membrane and matrix integrity [97]. 

The formation of new blood vessels involves not only cellular motility, growth, and sprouting, but also dynamic interactions with the endothelial basement membrane. Integrin-laminin interactions have been shown to regulate vessel branching [98] while recruited pericytes play an active role in vascular morphogenesis [99]. Matrix remodeling pathways are also highly involved in neovascularization, controlling neovessel growth, maturation, and regression during tissue repair [100]. Specific proteases such as MMP-9 and their inhibitors regulate major aspects of extracellular matrix turnover and degradation during vascular remodeling [101]. 

In addition to local effects at the injury site, MMP-9 has been shown to recruit EPCs from the bone-marrow [102] and can induce matrix release of vasculogenic cytokines including VEGF and TGF*β* [103, 104]. Studies using MMP-9 knockout mice have demonstrated that MMP-9 is essential for tumor vascularization [105] and augments EPC mobilization and migration in a hindlimb ischemia model [106]. Additionally, stem-cell-activating cytokines may be released from the extracellular matrix by MMPs [107], further potentiating the neovascularization process.

Other proteases implicated in vascular formation include membrane-type MMPs (MT-MMPs) that act on the matrix directly surrounding new vascular cells [108]. Other soluble proteases include MMPs-1, 2, 8, and 13 that are only activated in the extracellular matrix and degrade matrix components to enable neovessel growth [108]. MMPs are inhibited by mediators known as tissue inhibitors of MMPs (TIMPs) that highly regulate the breakdown of matrix. Additionally, cysteine proteases known as cathepsins and serine proteases have been shown to control blood vessel formation. These complex interactions between cells and their matrix help facilitate neovascularization from the initial mobilization of EPCs to their ultimate fate as neovessels [109, 110]. Together, these studies indicate that remodeling enzymes such as MMP-9 and others play a crucial role in vasculogenesis at both the injury site and in the bone-marrow where quiescent EPCs reside. 

### 3.8. TGF*β*

The TGF*β* superfamily consists of over 30 growth and differentiation factors that play vital roles in development and regulation of stem cell fate [111]. During wound healing, specific TGF*β* isoforms (*β*1, *β*2, and *β*3) are secreted as a complex with latent precursors that are modified in the extracellular space. TGF*β* is amongst the most well-studied signaling molecule in wound healing and is particularly linked to matrix and collagen production during wound healing. The ratio of expression of TGF*β*1 and TGF*β*3 is thought to regulate the ability of certain species and early gestation human fetuses to heal without scar [112].

In addition to its established role in fibrotic processes, TGF*β* has been linked to neovascularization pathways through multiple receptor and intracellular signaling mechanisms. For example, TGF*β* modulates vascular development by augmenting VEGF synthesis through Akt and ERK pathways [113]. TGF*β* has also been shown to activate the recruitment of VEGF-expressing hematopoietic effector cells, establishing a potent signaling network in the inflammatory wound environment that simultaneously stimulates neovascularization [114]. 

TGF*β* pathways can also act independently of VEGF. In an embryonic stem cell vasculogenesis model, TGF*β* was shown to stimulate neovessel growth via activin receptor-like kinase (ALK) receptors [115]. Moreover, TGF*β* can regulate non-endothelial cells during blood vessel maturation, specifically promoting vessel muscularization by stimulating MSC differentiation into pericytes [116]. TGF*β* can also activate the transdifferentiation of EPCs into myocytes (a process linked to pathologic intimal hyperplasia), highlighting the importance of tightly controlled cytokine pathways in vascular homeostasis [117].

Dysregulated TGF*β* signaling has been linked to vascular pathology in humans. Mutations in the human endoglin gene, a TGF*β* co-receptor, result in a vascular dysplasia known as hereditary hemorrhagic telangiectasia [118]. EPCs from these patients exhibit aberrant ALK signaling and impaired vascular tubule formation *in vitro*, suggesting that TGF*β* pathways are relevant to EPC function and vascular morphogenesis in humans. Collectively, these studies underscore the complex roles played by cytokines such as TGF*β* in activating EPCs and mesenchymal precursors to produce functional neovasculature.

## 4. Challenges for Translation 

**Figure 3:**
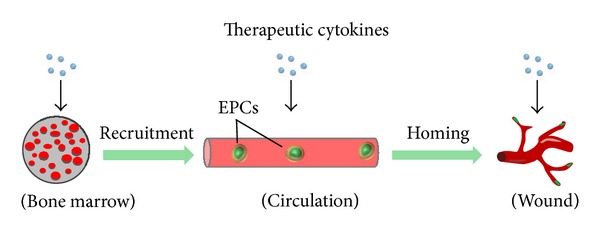
Cytokine-based approaches to augment vasculogenesis. Soluble molecules can be used to promote EPC production or activation from quiescent states in the bone-marrow during recruitment. Circulating EPCs can be targeted to injury sites via chemokines or modified at the cell surface level to promote egress from the circulation to the injury site during cell homing. Within the wound, EPC motility, proliferation, survival, and differentiation can be enhanced with cytokine therapies. Ultimately, a combination of cytokine cocktails, precise control of biochemical gradients, and modification of EPCs themselves may be needed to optimize vasculogenic therapies for clinical use.

A cascade of cytokines, growth factors, and other soluble mediators is released immediately following injury to orchestrate the repair of complex tissues [119]. Numerous *in vitro* and preclinical studies have demonstrated that cytokine-based therapies can have a profound and multifaceted effect on neovascularization and chronic wound healing [120] (Figure 3). Although most of these therapies remain unproven in controlled clinical trials, several recombinant cytokines have been shown to have a positive impact on nonhealing wounds.

Recombinant human PDGF (becaplermin) is approved by the US Food and Drug Administration for the topical treatment of lower extremity diabetic neuropathic ulcers. Although several randomized controlled studies have validated its efficacy for nonhealing wounds, it remains expensive and not widely utilized [121, 122]. Granulocyte-CSF (G-CSF) is another cytokine that has demonstrated clinical benefit for diabetic patients with foot infections. G-CSF limits the duration of antibiotic treatment, hospital length of stay, and rate of amputation [123, 124]. Despite small case reports suggesting its effectiveness for chronic ulcers [125–129], larger clinical studies are needed to determine its ability to enhance wound healing.

 Chemokine therapies are a promising strategy to promote neovascularization via modulation of the inflammatory response. Studies indicate that altered chemokine pathways may play a role in perpetuating the nonhealing nature of venous stasis ulcers [130]. These wounds may also have ineffective angiogenic drives, suggesting that molecular strategies capable of augmenting blood vessel formation may prove clinically successful [131]. For example, gene transfer of SDF-1 significantly enhanced EPC mobilization and vascularization in a hindlimb ischemia model, effects mediated through VEGF and nitric oxide synthase (NOS) [132]. In a similar model, VEGF-transduced EPCs significantly improved wound vascularity compared to control EPCs [133], suggesting that EPC-targeted approaches may be a feasible option for clinical therapy.

In addition to gene-based therapies, biomaterial delivery of vasculogenic cytokines has been shown to improve vasculogenesis during wound healing [134, 135]. Matrix components and spatial patterning can precisely regulate vasculogenic programs and have the potential to promote a richly vascularized repair environment [136, 137]. As these biomaterial and molecular technologies continue to advance, combination cytokine-EPC impregnated scaffolds may become a clinical reality. Currently, wound therapies targeting vasculogenic pathways are largely in the preclinical stage but we believe these evolving strategies will continue to represent a promising approach to chronic wound healing. 

## Figures and Tables

**Table 1:** Cytokines important in adult vasculogenesis.

Cytokine	Proposed vasculogenic mechanism
VEGF	Endothelial cell motility, proliferation, and survival
	EPC mobilization and homing
	Upregulation of other vasculogenic cytokines
	Promotes integrin expression

SDF-1	Trafficking of EPCs and HSCs
	Hypoxia-responsive EPC recruitment

PDGF	Pericyte recruitment and vessel maturation
	EPC migration and expansion
	Closely associated with VEGF pathways

FGF	VEGF-dependent neovascularization
	Bone-marrow-derived perivascular cell recruitment
	Vascular remodeling

GM-CSF	EPC recruitment and mobilization
	Monocyte/macrophage recruitment and activation
	Modulation of immune and inflammatory pathways

S1P	Promotes migration of embryonic angioblasts and endothelial cells
	Blood vessel maturation
	May augment vasculogenic effects of PDGF

MMP-9	EPC recruitment and mobilization
	Induces release of vasculogenic growth factors from the extracellular matrix

TGF*β*	Promotes VEGF pathways
	Enhances MSC differentiation into pericytes
	Activates EPC transdifferentiation into smooth muscle
